# Prevention of sepsis by correcting apoptosis

**DOI:** 10.1186/cc11946

**Published:** 2013-03-19

**Authors:** M Puhtinskaya, V Estrin

**Affiliations:** 1 Research Institute of Obstetrics and Pediatrics, Rostov-on-Don, Russia

## Introduction

Activation of apoptosis in lymphocytes determines the development of neutropenia and of sepsis [1,2]. We investigated prevention of sepsis and correction of lymphocyte apoptosis by recombinant human granulocyte colony-stimulating factor (hr-GCSF, fillgrastim) [1,2].

## Methods

With the permission of the ethics committee, a controlled, randomized, blind clinical trial included 69 term newborns on mechanical ventilation, without neutropenia and clinical signs of infection, with a content of lymphocytes in early apoptosis (AnnexinV-FITC+PI-) of >9.59%, and in late (AnnexinV-FITC+PI+) of 0.56%. Lymphocytes in apoptosis were detected using antibodies to AnnexinV and propidium iodide staining method of immunophenotyping (flow cytometry; Beckman Coulter Epics XL, USA). The survey was conducted at admission, at 3 to 5 days, and 20 days. The method of random numbers in Group I included 39 newborns who on admission (with written parental consent) received an intravenous infusion of hr-GCSF dose of 10 μg/kg, 3 days. Newborns of Group II (*n *= 30) did not receive hr-GCSF. Power of the study was 80% (α ≤ 0.05).

## Results

For 3 to 5 days, Group I significantly decreased apoptosis of lymphocytes in the early from 16.1% to 7.8%, and in late from 1.3% to 0.1%. The development of sepsis and neutropenia have been reported. We observed no clinical or laboratory signs of adverse effects of the drug. Fatal outcomes (*n *= 4) are not associated with hr-GCSF, which was confirmed postmortem. Decreased duration of mechanical ventilation (*P <*0.05). In Group II, 27 patients at 3 to 5 days developed neutropenia and increased lymphocytes in apoptosis (*P <*0.05). Sepsis was diagnosed in 19 children; eight fatal outcomes.

## Conclusion

hr-GCSF reduces the incidence of septic complications and one of the mechanisms of its clinical effectiveness is the reduction of apoptotic factors affecting the development of neutropenia.
